# Maternal Vibration: An Important Cue for Embryo Hatching in a Subsocial Shield Bug

**DOI:** 10.1371/journal.pone.0087932

**Published:** 2014-01-31

**Authors:** Hiromi Mukai, Mantaro Hironaka, Sumio Tojo, Shintaro Nomakuchi

**Affiliations:** 1 The United Graduate School of Agricultural Sciences, Kagoshima University, Kagoshima, Japan; 2 Department of Biology, Faculty of Medicine, Hamamatsu University School of Medicine, Hamamatsu, Japan; 3 Department of Applied Biological Sciences, Faculty of Agriculture, Saga University, Saga, Japan; University of Tours, France

## Abstract

Hatching care has been reported for many taxonomic groups, from invertebrates to vertebrates. The sophisticated care that occurs around hatching time is expected to have an adaptive function supporting the feeble young. However, details of the characteristics of the adaptive function of hatching care remain unclear. This study investigated the hatching care of the subsocial shield bug, *Parastrachia japonensis* (Heteroptera: Parastrachiidae) to verify its function. Results show that the *P. japonensis* mothers vibrated the egg mass intermittently while maintaining an egg-guarding posture. Then embryos started to emerge from their shells synchronously. Unlike such behaviors of closely related species, this vibrating behavior was faint, but lasted more than 6 h. To investigate the effect of this behavior on hatching synchrony and hatching success, we observed the hatching pattern and the hatching rate in control, mother-removed, and two artificial vibration groups. Control broods experienced continuous guarding from the mother. Intermittent artificial vibration broods were exposed to vibrations that matched the temporal pattern of maternal vibration produced by a motor. They showed synchronous hatching patterns and high hatching rates. However, for mother-removed broods, which were isolated from the mother, and when we provided continuous artificial vibration that did not match the temporal pattern of the maternal vibration, embryo hatching was not only asynchronous: some embryos failed to emerge from their shells. These results lead us to infer that hatching care in *P. japonensis* has two functions: hatching regulation and hatching assistance. Nevertheless, several points of observational and circumstantial evidence clearly contraindicate hatching assistance. A reduction in the hatching rate might result from dependence on maternal hatching care as a strong cue in *P. japonensis*. We conclude that the hatching care of *P. japonensis* regulates the hatching pattern and serves as an important cue to induce embryo hatching.

## Introduction

Generally, in contrast to more developed stages, embryos and newly hatched young have undeveloped locomotor and sensory organs. Consequently, they are vulnerable to dangerous and changeable environments. Particularly during hatching, when embryos emerge from their egg capsules, simultaneous changes in the physical environment and their developing status, and their biotic interactions present the highest risk for feeble embryos [1]. Therefore, animal parents of several species have used special care during the hatching moment, i.e. ‘hatching care’, to increase their offspring's chances of survival. Hatching care has been reported in many taxonomic groups from invertebrates to vertebrates. For example, parents in most avian species [2]–[5] and *Crocodylus* [6], [7] actively peck or bite eggs at the time of hatching to break the hard shell. Additionally, various parental hatching care behaviors have been reported for arthropod species. Mothers of some species of subsocial spiders break into the egg sac at hatching [8]–[10]. Most crustacean parents generate active abdomen pumping. Synchronous larval release occurs soon after the behavior [11], [12]. The male giant water bug, *Lethocerus deyrolli*, splashes water on the mature egg mass immediately before synchronous hatching [13].

Such complex and sophisticated hatching care in animals is readily expected to have the adaptive function of supporting the feeble young. However, details of the contents of the adaptive function of hatching care––who benefits from care and how––remain unclear. Recently, we proposed that parental hatching care behaviors are classifiable into two groups related to adaptive functions as ‘hatching assistance’ and ‘hatching regulation’ correspond to the hatching care of a stinkbug [14]. Subsocial burrower bug *Adomerus rotundus* mothers make physical vibrations while holding the egg mass. To elucidate the functions of hatching care in a previous study, we specifically addressed the effects of care-removal on the hatching rate and hatching pattern. For the brood that had been isolated from the mother hatched asynchronously, however, the total hatching rate was not influenced by the mother removal treatment. Based on these results, we concluded that maternal vibration of *A. rotundus* has the function of hatching regulation, which acts to synchronize the hatching pattern, rather than hatching assistance, which acts to improve the hatching success of eggs [14]. Based on our attempt, we must survey more instances of hatching care to ascertain whether our definitions of the functions and verification methods are appropriate.

A subsocial shield bug, *Parastrachia japonensis* (Heteroptera: Parastrachiidae) is a subsocial heteropterans and closely related species of *A. rotundus*. In addition, *P. japonensis* mothers show complex maternal care including egg guarding, production of trophic eggs, protection of nymphs, and progressive provisioning [15]–[18]. The hatching and the developmental stages of siblings are also synchronized remarkably, as are those of *A. rotundus* [18], [19]. Preliminary observations reveal that the embryos of *P. japonensis* cannot hatch when the mother is removed from the egg mass [16]. According to these observations, functions of the hatching care of *P. japonensis* were expected to be ‘hatching assistance’ and to differ greatly from that of *A. rotundus*. Comparison of these species contributes not only to clarification of the diversity of hatching care among the closely related species, but also to the proposal of more precise definitions and verification methods. This study investigated the behavioral features of hatching care and revealed its adaptive function in the subsocial shield bug, *P. japonensis*.

## Materials and Methods

### Insect Sampling and Rearing

We collected *P. japonensis* mothers that were guarding their egg masses at a field site, Hinokuma-yama, a small forested hill in Saga Prefecture, Japan (33°16′N, 130°16′E), during June 2010. No specific permit was required for these studies. The location is not privately owned or protected in any way. The studies did not involve endangered or protected species.

We reared egg-guarding mothers individually in clear plastic cups (8 cm diameter; 4 cm height) lined with substrate (soil and fallen leaves) for 1 week at 22°C under a long-day regime of 16 h light and 8 h dark in an incubator, but all subsequent observations and experiments were conducted at 25°C. A plastic lid was used to cover the cups to keep the bugs from escaping. We turned the light source in the incubator on at 600 h and off at 2200 h to simulate the appropriate seasonal outdoor conditions.

### Observations of vibrating behavior

To monitor maternal behavior at the time of hatching, we observed seven mothers holding a mature egg mass, which turned pink and which developed conspicuous eye-spots immediately before hatching. To facilitate the observations, we transferred each mother with its egg mass to a clear plastic box (1.5 cm×5.5 cm×8.5 cm). All mothers with egg masses were filmed from the side of the box using a video camera (HDR-XR500V; Sony Corp.) mounted on a tripod. Video monitoring was continued for 6 h from the hatching. These observations revealed that all mothers showed a physical vibration at the time of nymphal hatching. In addition, we recorded the vibrational behavior of three mothers with a high-speed camera (Exilim EX-F1; Casio Computer Co. Ltd.) so that detailed patterns of the vibration can be examined further. We started to film them just before the vibration would occur and recorded the mother's motion for 5–10 s (300 frames/s; fps). From the video data, we quantified the vibration pattern to a millimeter compared with the known body length of mothers.

### Effects of maternal care

To verify whether the maternal vibration served to synchronize hatching or not, we monitored the temporal pattern of hatching of four groups: a control group, a mother-removed group, and two artificial vibration groups. Mothers in the control group (*N* = 8) were not disturbed. They were left with their egg masses. It was observed that the control mothers suspended the egg mass with the proboscis at the moment of hatching. In the mother-removed group (*N* = 7), mothers were removed from the egg masses before hatching. Hosokawa et al. (2012) reported that mothers of *P. japonensis* start to excrete a copious amount of symbiont-containing white mucus from the anus onto the egg mass at around 45 min before egg hatching. At about 5 min after the end of excretion, the embryos started to hatch synchronously [19]. Therefore, we isolated mature egg masses from mothers immediately after they finished the excretion behavior. Then, we put a fine nylon thread through the midline of each egg mass and artificially suspended it by holding it with a clip in the experimental plastic box.

In the artificial vibration group, we set following two groups: intermittent artificial vibration and continuous artificial vibration. We isolated egg masses after the excretion, and suspended them by holding them with a clip. We fixed an electrodynamic cordless motor (pellet pestle cordless motor; Kimble Chase Life Sciences and Research Products LLC), which operates at 2000–3000 rpm with fresh batteries and no load, lightly on the nylon thread at the position of 3 cm from an egg mass. For the intermittent vibration group (*N* = 7), we provided an artificial vibration using a cordless motor during 15 min from the end of mucus excretion. From our observations, we confirmed that the mothers vibrated, on average, one time per 30 s during the first 15 min. Furthermore, from the analysis by using high-speed photography, we revealed that *P. japonensis* mothers move their abdomen only a few mm vertically slightly up and down within 1 s. Therefore, to expose the mature egg mass to this frequency of artificial vibration, we switched on once for an instant every 30 seconds with the cordless motor attached to the end of the nylon thread. For the continuous vibration group (*N* = 5), we provided an artificial vibration continuously during 15 min from the end of excretion. We kept the electrodynamic cordless motor adhered to the nylon thread during this period. In these experimental groups, there was no attempt to match the frequency content of individual vibrations.

We counted the hatchlings in each group; then we calculated the hatching rate. Counts were conducted every 5 min during the first 90 min; then we counted them once again at 24 h to ascertain whether the maternal vibrations had improved the hatching success or not. To avoid double-counting, counted hatchlings were removed to another container using an insect aspirator. To confirm whether the remaining eggs hatched later, unhatched eggs at 24 h of the several egg masses in each group were kept in the incubator.

### Statistical Analyses

All statistical analyses were conducted using R, ver. 2.9 (R Development CoreTeam, 2009). To ascertain whether the hatching occurrence varied significantly among the four groups (control, mother-removed, intermittent artificial vibration, and continuous artificial vibration), the respective points at which 50% of a clutch had hatched in each of the four groups was compared statistically using the nonparametric Wilcoxon rank-sum test. We adjusted the pairwise significance level (type 1 error: α) to 0.008 (0.05/6) using Bonferroni correction. We also analyzed the hatching success of the eggs at 24 h after hatching using the nonparametric Wilcoxon rank-sum test to verify the effect of maternal vibration on hatching success among the four groups. We adjusted the pairwise significance level (type 1 error: α) to 0.008 (0.05/6) using Bonferroni correction.

## Results

### Hatching process and vibrating behavior by the mother

**Figure 1:**
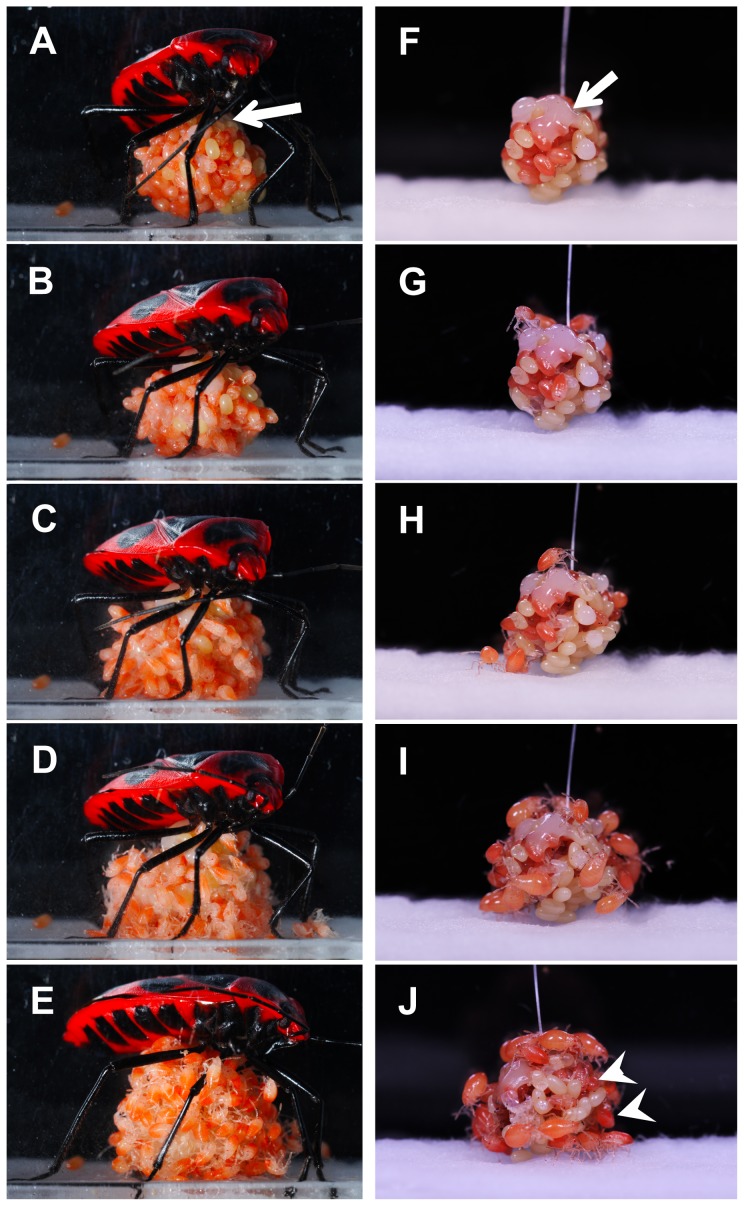
Hatching processes of the control and the mother-removed egg mass. After mucus excretion, the mother suspended her own egg mass with her proboscis and maintained the egg-guarding posture (A). Mothers slightly vibrated the egg masses maintaining the guarding posture; then fertile eggs started to hatch synchronously approximately 4 min later (B). The embryos, moving peristaltically, emerged gradually from their shells (C). Almost all embryos had emerged completely by 30 min later (D). Newly hatched nymphs gradually began to move on the mass of empty eggshells. Then they started to take up the mucus secretion and the trophic eggs on the mass (E). However, if the mothers were removed from the egg mass, then about half the number of embryos failed to hatch. The other half hatched asynchronously for 24 h (F–J). Arrows indicate mucus secretions on the egg mass (A, F). Arrowheads indicate nymphs that are still hatching or which remain in the eggshell (J).

Video monitoring of egg-guarding bugs revealed that all mothers (*N* = 7) showed a faint vibration (Video S1). Before the start of hatching, the maternal excretion behavior continued for approximately 52.0±26.1 min (mean ± SD, *N* = 7). After the excretion behavior, the mothers lifted the egg mass using the proboscis and resumed the egg-guarding posture (Fig. 1A). At 4.7±2.8 min later, mothers started body shaking.

**Figure 2:**
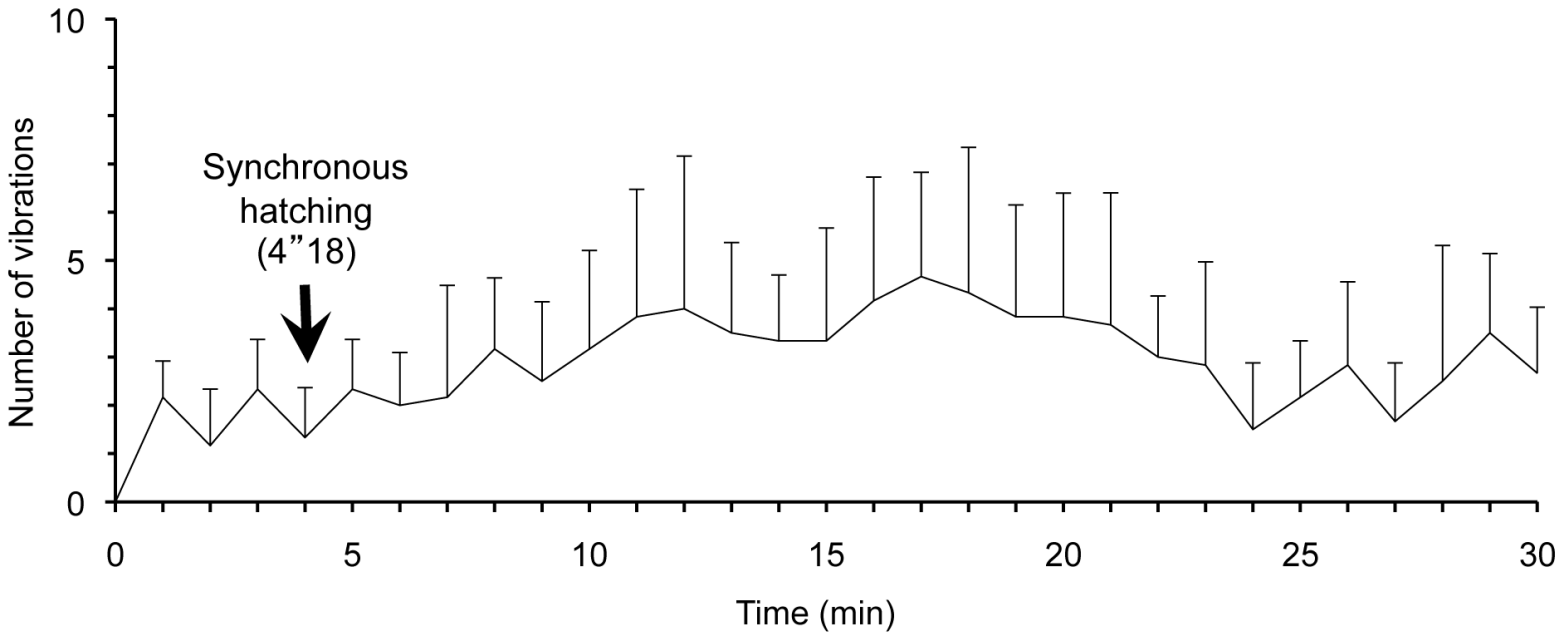
Occurrence pattern of maternal vibration. Means + SD are shown for numbers of vibration/min from the start of vibration generated by seven mothers. Arrows indicate the beginning of synchronous hatching.

Mothers vibrated a suspended egg mass while maintaining the egg-guarding posture. Mothers showed intermittent vibrating behavior, i.e., they did not move for several tens of seconds after the prior vibration; then they vibrated their body only once. Observation using a high-speed photography revealed that maternal vibration comprised only a faint single vertical motion, moving the abdomen 2–3 mm vertically slightly up and down. The single vertical motion took approximately 0.3 s to move the mother's abdomen (*N* = 3). To quantify the temporal pattern of the maternal vibration, we counted the abdominal movements per minute for 30 min from the beginning of the vibrating behavior (Fig. 2). Mothers showed, on average, a total of 87.5±1.8 times (mean ± SD, *N* = 7), constituting 2.9±1.8 times (mean ± SD, *N* = 7) per minute of vibration during this period. Although no distinct peak of the maternal vibration or remarkable change of the mother's behavior was observed, highly synchronous hatching began at 4.3±1.8 min (Figs. 1B–1C, mean ± SD, *N* = 7) from the beginning of vibration.

At 30 min after the start of hatching, 90.3±7.3% (mean ± SD, *N* = 7) of the embryos emerged from their shells (Fig. 1D). Newly hatched nymphs remained motionless for a few minutes, but they began to move gradually. Then they started to walk around actively and aggregated on the egg mass within 5 min. The newborn nymphs took up the mucus secretion and the trophic eggs with their respective proboscises immediately (Fig. 1E). Some mothers continued the vibrating behavior for more than 6 h from the beginning of vibration.

### Effects of maternal care on the hatching pattern

**Figure 3:**
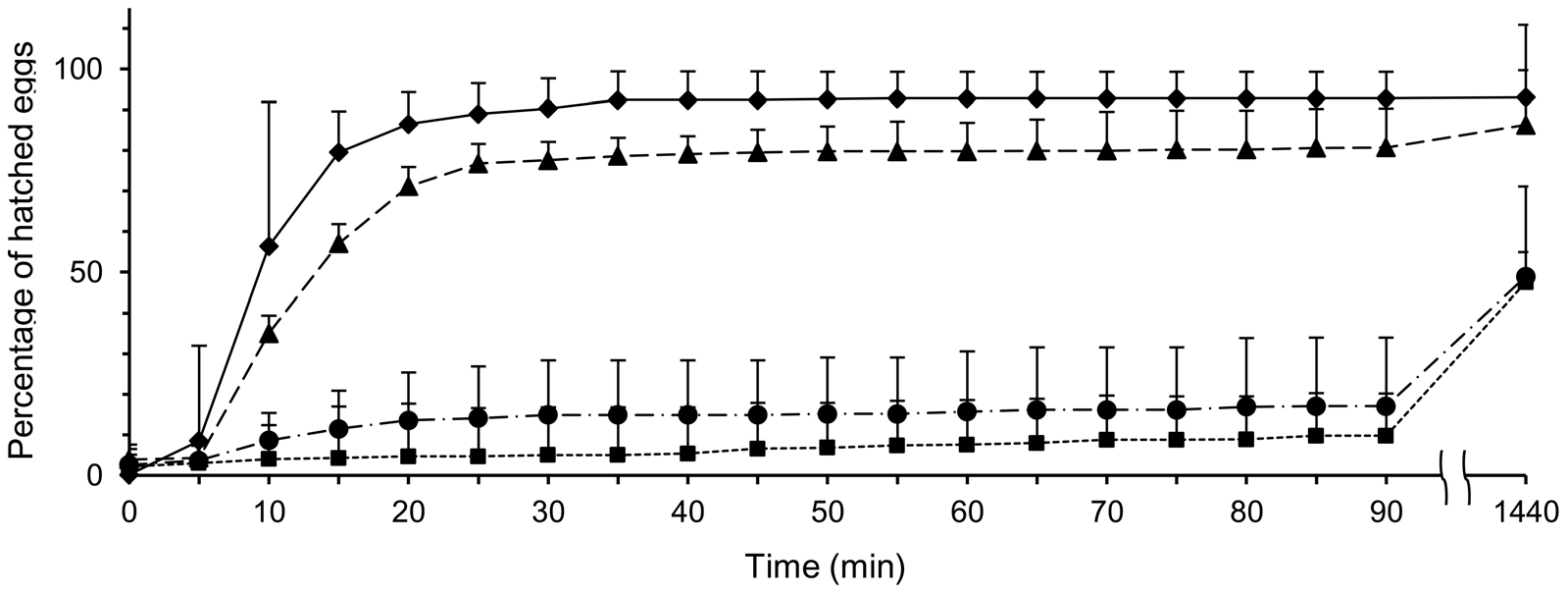
Hatching pattern of embryos exposed to the different treatments. Hatching patterns of the control group (solid line and diamonds, *N* = 8), mother-removed group (dotted line and squares, *N* = 7), intermittent artificial vibration group (dashed line and triangles, *N* = 7), and continuous artificial vibration group (dotted-dashed line and circles, *N* = 5). Means + SD are shown for the percentage of hatched eggs at 5 min intervals for the first 90 min, and for 24 h from the start of hatching.

We compared hatching rates among the four groups: control group, mother-removed group, and two artificial vibration groups. In the control group, hatching occurred synchronously: 90.3±7.3% (mean ± SD, *N* = 8) of the embryos had completed hatching within the first 30 min (Fig. 3). If the mothers were removed from the egg mass, then the aspects of the hatching changed drastically. About half of the embryos failed to hatch; the other half hatched asynchronously (Figs. 1F–1J). In the mother-removed group, embryos took a long time to hatch, with 5.0±4.5% (mean ± SD, *N* = 7) of the embryos hatching within the first 30 min. When we exposed egg masses to artificial vibration, the embryos began to hatch rapidly one after another during approximately 5 min after we started the vibration. The average percentage of hatched eggs ± SD was 77.6±11.8% within the first 30 min. However, when we exposed egg masses to continuous artificial vibration, embryos took a long time to hatch, with 14.9±13.4% (mean ± SD, *N* = 5) of the embryos hatching with the first 30 min.

The point at which 50% of the eggs of the clutch had hatched in the control group was significantly earlier than in the mother-removed group (Wilcoxon rank-sum test: *z* = −3.31, *N*_1_ = 8, *N*_2_ = 7, *P*<0.001, Bonferroni corrected *α* = 0.008), and in the intermittent artificial vibration group was earlier than in the mother-removed group (*z* = −3.22, *N*_2_ = 7, *N*_3_ = 7, *P* = 0.0013, Bonferroni corrected *α* = 0.008) and was later than in the control group (*z* = −2.80, *N*_1_ = 8, *N*_3_ = 7, *P* = 0.0050, Bonferroni corrected *α* = 0.008). However, the point at which 50% of the eggs of the clutch had hatched in the continuous artificial vibration group occurred later than in the control group (*z* = 2.77, *N*_1_ = 8, *N*_4_ = 5, *P* = 0.003, Bonferroni corrected *α* = 0.008) and in the intermittent artificial vibration group (*z* = 2.33, *N*_3_ = 7, *N*_4_ = 5, *P* = 0.004, Bonferroni corrected *α* = 0.008). However, no significant difference was found between the mother-removed group and the continuous artificial vibration group (*z* = 4.0, *N*_2_ = 7, *N*_4_ = 5, *P* = 0.77, Bonferroni corrected *α* = 0.008).

### Effects of maternal care on hatching success

We compared hatching success among the four groups: control group, mother-removed group, and two artificial vibration groups. The rates of embryos in the identical egg mass hatching at 24 h from the start of hatching in the control group were 93.0±6.7% (mean ± SD, *N* = 8). However, that in the mother-removed groups reached only 47.5±24.6% (mean ± SD, *N* = 7). In the intermittent artificial vibration group, 86.3±7.5% of all embryos hatched during 24 h from the start of hatching (mean ± SD, *N* = 7). The rates of embryos in the identical egg mass hatching at 24 h from the start of hatching in the control group was significantly higher than in the mother-removed group (Wilcoxon rank-sum test: *z* = 6.13, *N*_1_ = 8, *N*_2_ = 7, *P* = 0.001, Bonferroni corrected *α* = 0.008), and in the continuous artificial vibration group (*z* = 5.8, *N*_1_ = 8, *N*_4_ = 5, *P* = 0.007, Bonferroni corrected *α* = 0.008). However, no significant difference between the control group and the intermittent artificial vibration group (*z* = 5.1, *N*_1_ = 8, *N*_3_ = 7, *P* = 0.18, Bonferroni corrected *α* = 0.008), and between the mother-removed group and the continuous artificial vibration group (*z* = 3.7, *N*_2_ = 7, *N*_4_ = 5, *P* = 0.87, Bonferroni corrected *α* = 0.008). The rates of embryos in the identical egg mass hatching at 24 h from the start of hatching in the intermittent artificial vibration group was significantly higher than in the continuous artificial vibration group (*z* = 5.2, *N*_1_ = 8, *N*_3_ = 7, *P* = 0.18, Bonferroni corrected *α* = 0.008).

The embryos that had not hatched during the observation never hatched thereafter. All blackened and died.

## Discussion

### Features of vibrating behavior

We discovered that *Parastrachia japonensis* mothers show characteristic hatching care. They vibrate a suspending egg mass by moving their abdomens immediately before hatching. Approximately 5 min after the vibration started, almost all embryos hatched synchronously. Earlier studies of hatching cues have shown that hatching induced by vibrational stimulation is widespread both in social species that provide hatching care, and in nonsocial species that do not. For instance, in some nonsocial reptilian [20] and amphibian species [21], embryos in the egg clutch can assess vibrational cues from siblings or predators, and respond to hatching. Additionally in some social arthropod species, especially some Crustaceans, mothers generate a vibrational stimulation used as the signal for embryo hatching [11], [12], [22]. Our previous reports explained that mothers of *Adomerus rotundus*, a closely related species of *P. japonensis*, display a physical vibration that coincides with the time at which most embryos hatch [14]. However, although both the hatching care of *P. japonensis* and *A. rotundus* include vibration of their egg masses, some important differences are apparent in the features of the vibrating behavior.

The notable one is the occurrence pattern of vibrating behavior. In *A. rotundus*, the maternal vibration increased initially over time, reaching a peak at approximately 9 min after the start of vibration when most of the eggs hatched. This vibrating behavior lasted about 23 min. However, in *P. japonensis*, the maternal vibration did not have a distinct peak. Vibrating behavior of *P. japonensis* lasted interminably over 6 h from the beginning of vibration, even after almost all embryos' hatching had already finished (Fig. 2). Detailed temporal patterns of vibration were also different in these two species. In *A. rotundus*, maternal vibration apparently took place intermittently with alternate occurrence of two conspicuous periods: a vibration period and a pause period. The vibration period included several pulses, which comprised the single vertical motion of the vibrating mother's body. While in *P. japonensis*, mothers provided a faint vibration to the suspended egg mass, i.e., mothers vibrated their bodies only once per several tens of seconds (Video S1). Furthermore, we are confident that the vibration intensity differs among species by observation using high-speed photography. The rough vibrating behavior in *A. rotundus* is readily detectable by the human eye, although that in *P. japonensis* is difficult to observe.

Why were these differences observed in closely related species? Among closely related species of some crabs, of which mothers generate abdominal pumping at hatching, some comparisons have been made of the frequency and the occurrence pattern of maternal pumping behavior [23]–[25]. The differences of the hatching care are presumably attributable to the physiological constraints of eggs, such as their respective sizes [25]. Considering that, differences between *P. japonensis* and *A. rotundus* might be derived from physiological constraints. To elucidate the diversity of the stimulus in hatching care, future studies should examine more details of the physiological and ecological constraints of these species and other subsocial bugs practicing complex maternal care resembling that of *A. rotundus* and *P. japonensis* provided by mothers [26]–[29].

### Adaptive functions of maternal vibration

A previous report described the functions of hatching care as classifiable into two groups: ‘hatching assistance’ and ‘hatching regulation’ [14]. Hatching assistance, a form of parental care performed by parents to provide some assistance for their young, enhances hatching success, as shown by parents of most avian species: they break into the hard egg shell to assist their young [2]–[5]. Hatching regulation is a form of parental care that is performed by parents to provide some physical or chemical stimulation for embryos to regulate the hatching pattern, as seen in subsocial spiders: mothers adjust the egg-hatching time in response to the threat of predation [30]. According to this definition, it can be inferred that *P. japonensis* acquired both functions: hatching assistance and hatching regulation. However, the hatching care of *P. japonensis* is evidently different from other examples of hatching assistance in avian, crocodilian, and arachnid species. In *P. japonensis*, mothers give only a faint vibration to the egg mass. In fact, even when we provided continuous artificial vibration that did not match the temporal pattern of maternal vibration for the isolated egg mass, the hatching tended to fail and to occur asynchronously (Fig. 3). This observation revealed that the physical vibration does not induce shell destruction.

In contrast, results strongly suggest that hatching care of *P. japonensis* serves as hatching regulation because the maternal vibration also influences hatching synchronization. The hatching pattern in the control group is extremely synchronized, but it is asynchronous in the mother-removed group. In addition, in the intermittent artificial vibration group that matched the temporal pattern of maternal vibration, hatching occurred with comparable synchrony of hatching in the control group. The ecological background of *P. japonensis* also supports our claim that hatching care of this species acts as hatching regulation. The adaptive functions of the synchronous hatching and the necessity of the hatching regulation of *A. rotundus*, which were discussed in a previous report, such as ‘the risk of cannibalism’, ‘defense against predators’ and ‘facilitate the more effective provision of care’ [14], are true also of *P. japonensis* [16]–[18], [31]. In addition, the vibrating behavior of *P. japonensis* might have other functions for hatched nymphs such as maintaining a large nymphal aggregation, or to activate taking up of the mucus secretion, which might be involved in uric acid recycling during the diapause period [19], [32], [33]. These results suggest that the hatching care of *P. japonensis* serves as hatching regulation.

Furthermore, we found that the temporal pattern of vibration is an extremely influential factor to hatching in *P. japonensis*. When we provided the intermittent artificial vibration, almost all embryos could hatch successfully and synchronously, although the point at which 50% of eggs of the clutch had hatched was slightly later than that in the control group. On the other hand, interestingly, when we provided continuous artificial vibration, embryo hatching was not only asynchronous but also some embryos failed to emerge from their shells. We indicated that even such a playback simply imitating the temporal pattern of the vibration can regulate the hatching pattern and work as an effective cue to induce embryo hatching. The results also showed that there is a significant difference in the hatching pattern between the real vibrations produced by mothers and artificial vibrations produced by the motor. In the present situation, we cannot explain this correctly. We, however, thought that the actual maternal vibration would contain some different major influential elements possibility such that the acceleration, frequencies content, or fine-scale temporal pattern could be included in the maternal signals. Further studies are needed to identify the key element of the vibrational traits more accurately, or to detect other factors such as pheromones from a living mother influencing hatching.

### Necessary cue for embryo hatching

Hatching regulation is a form of parental care that regulates the temporal pattern of hatching to adapt to the needs of hatchlings. According to this definition, all embryos are expected to be able to hatch independently, without physical or chemical assistance from their parents. Nevertheless, nearly 60% of the embryos of *P. japonensis* failed to hatch when we intercepted the maternal vibration and when we provided continuous artificial vibration that did not match the temporal pattern of maternal vibration for the isolated egg mass. How do we interpret that discrepancy?

Hatching systems related to hatching cues are classifiable into two groups: ‘spontaneous hatching (SH)’ and ‘environmentally cued hatching (ECH)’. ‘Fixed hatching’ or ‘normal hatching’ is synonymous with spontaneous hatching used in hatching studies of amphibians [1] or reptilians and avians [34]. SH is defined as a hatching system that operates without an external cue [1], [35], [36]. SH occurs at a consistent stage in development, or potentially, after a consistent embryonic period, irrespective of environmental conditions. In contrast, ECH is defined as a “decision” based on information. It might depend on a specific behavioral or physiological process that is cued environmentally [1]. For an animal species that depends entirely on an SH system, or on both SH and ECH, embryos hatch with no stimulation. In a species depending solely on ECH, hatching will not occur without the cue. Embryos die when the energy reserves become exhausted. For example, in some solitary zooplankton *Daphnia* species, embryos never hatch without moisture stimulation [37]. Probably, the hatching system of *P. japonensis* is a type of ECH only. Therefore, interception of the maternal vibration or providing continuous artificial vibration might affect the hatching success.

This study revealed that the previous definition of ‘hatching assistance’ is inappropriate. Hatching success can be expected to be influenced not only by hatching assistance, but also by a hatching system. Therefore, verifying the effect of maternal care solely by a cue deprivation experiment is reckless, although it is a simple and good method for classifying the hatching care, because in some species depending only on ECH, the care itself can constitute a cue that induces hatching. The hatching will never occur without providing the care in a case where the cue is necessary for post-hatched offspring because the survival rate of new hatchlings is reduced extensively even if they have hatched successfully. Based on the discussion presented above, the form of hatching care can be re-defined: ‘Hatching assistance’ is a form of parental care by which parents assist hatching by reducing the physical burden of embryos. ‘Hatching regulation’ is a form of parental care by which the parents regulate the temporal pattern of hatching without reducing the burden. We have classified the function of hatching care in accordance with ascertaining whether parental care served in previous studies (1) to improve the hatching success and (2) to influence to the hatching pattern. Therefore, in addition to these indexes, we must incorporate consideration of a new index: (3) how parental care is related to embryo hatching.

In this paper, we proposed and assessed a more appropriate definition and verification methods of functions related to hatching care than those reported in the literature [14]. Our proposals are expected to clarify previous and future studies of parental hatching care. Future studies are expected to extend the classification of hatching care, and to elucidate the evolutionary background of hatching care and parent–embryo interactions.

## Supporting Information

Video S1 **Maternal vibrating behavior and hatching process of** ***Parastrachia japonensis*** **.** The video presents three phases of the hatching process. At the beginning of the vibration behavior, a mother suspends the unhatched egg mass coated by mucus excretion (during 0–20 s in video data). At 4 min later, embryos are hatching synchronously from an egg mass (during 21–40 s). Around 15 min later, newly hatched nymphs are walking on the empty eggshells to take up mucus secretion and trophic eggs (during 41–60 s). In all three phases, only one vibrating behavior of the mother is apparent.
(WMV)
